# Supplementary material for: Predicting the Impact of Diffuse Alveolar Damage through Open Lung Biopsy in Acute Respiratory Distress Syndrome—The PREDATOR Study
Source: J Clin Med. 2019 Jun 11;8(6):829. doi: 10.3390/jcm8060829 (PMC6616523; doi:10.3390/jcm8060829)
Supplement: Supplementary file 1 [file jcm-08-00829-s001.zip › Article Predator_Table S1_V7 variables recorded.docx]

**Table S1: Variables recorded in the case record form**

**Comorbidities** (yes /no)

Active smoking Active solid neoplasms

Active hemato-oncology neoplasm Chemotherapy within the last 3 month

Organ transplant Chronic obstructive pulmonary disease

Acute immunodeficiency syndrome/human immunodeficiency virus

Arterial hypertension Coronary ischemic

Chronic cardiac failure (NYHA III or IV) Chronic kidney injury

Diabetes mellitus Diabetes mellitus requiring insulin

**Chronic medication** (yes/no)

Domiciliary oxygen therapy Inhaled β2 agonist Inhaled steroids

Systemic steroids

**Baseline characteristic**

Gender (female/male) Age (years)

Weight (Kg) Date of hospital admission

Date of ICU admission Date of ARDS diagnosis

Date of open lung biopsy Date of hospital discharge

**Variables recorded at the day of ARDS diagnosis and open lung biopsy perform**

FiO2 (%) PaO2 (mmHg)

PaCO2 (mmHg) Tidal volume (ml and ml/kg measured body weight))

Plateau pressure (cmH2O) PEEP (cmH2O)

Arterial pH Respiratory rate (breaths per minute))

Heart rate (beats per minute) Systolic arterial pressure (mmHg)

Temperature (°C) International normalized ratio

Prone position (yes/no) Hemoglobin (gr/dl)

Leukocytes (cells/microl) Platelets (cells/microl)

Creatinine (mg/dl) Intravenous steroids (yes/no)

Total bilirubin (mg/dl) Antiviral (yes/no)

Norepinephrine (mcg/kg/min) Antibiotics (yes/no)

Antifungal (yes/no)

Inhaled steroids (yes/no)
