# Supplementary material for: Predicting the Impact of Diffuse Alveolar Damage through Open Lung Biopsy in Acute Respiratory Distress Syndrome—The PREDATOR Study
Source: J Clin Med. 2019 Jun 11;8(6):829. doi: 10.3390/jcm8060829 (PMC6616523; doi:10.3390/jcm8060829)
Supplement: Supplementary file 1 [file jcm-08-00829-s001.zip › Article Predator_Table S2_V7 missing data.docx]

**Table S2:** Missing data in trainee and validation cohorts

|  | Trainee cohort  (n=193) | Validation cohort  (n= 65) | Percentage difference between groups |
| --- | --- | --- | --- |
| **Day of ARDS** | | | |
| FiO2 | 20(10) | 3(5) | 5 |
| PaO2 | 20(10) | 4(6) | 4 |
| PaCO2 | 37(19) | 8(12) | 7 |
| Tidal volume | 77(40) | 23(35) | 5 |
| Plateau pressure | 131(68) | 49(75) | -7 |
| PEEP | 58(30) | 18(28) | 2 |
| Driving pressure | 132(68) | 49(75) | -7 |
| Static compliance | 141(73) | 50(77) | -4 |
| Arterial pH | 37(19) | 9(14) | 5 |
| Number of affected quadrants in the chest X ray | 83(43) | 28(43) | 0 |
| Respiratory rate | 21(11) | 4(6) | 5 |
| Heart rate | 56(29) | 18(28) | 1 |
| SAP | 68(35) | 20(31) | 4 |
| Temperature | 68(35) | 19(29) | 6 |
| Hemoglobin | 44(23) | 13(20) | 3 |
| Leukocytes | 44(23) | 13(20) | 3 |
| Platelets | 40(21) | 11(17) | 4 |
| Creatinine | 41(21) | 11(17) | 4 |
| INR | 60(31) | 18(28) | 3 |
| Total bilirubin | 75(39) | 21(32) | 7 |
| Dobutamine | 128(66) | 48(74) | -8 |
| Norepinephrine | 67(35) | 19(29) | 6 |
| **Day of OLB** | | | |
| FiO2 | 20(10) | 3(5) | 5 |
| PaO2 | 19(10) | 3(5) | 5 |
| PaCO2 | 21(11) | 4(6) | 5 |
| Tidal volume | 33(17) | 8(12) | 5 |
| Plateau pressure | 96(50) | 29(45) | 5 |
| PEEP | 5(3) | 1(2) | 1 |
| Driving pressure | 100(52) | 29(45) | 7 |
| Static compliance | 100(52) | 29(45) | 7 |
| Arterial pH | 21(11) | 4(6) | 5 |
| Number of affected quadrants in the chest X ray | 90(47) | 28(43) | 4 |
| Respiratory rate | 22(11) | 5(8) | 3 |
| Heart rate | 52(27) | 16(25) | 2 |
| SAP | 64(33) | 19(29) | 4 |
| Temperature | 53(27) | 16(25) | 2 |
| Hemoglobin | 56(29) | 18(28) | 1 |
| Leukocytes | 57(30) | 18(28) | 2 |
| Platelets | 23(12) | 6(9) | 3 |
| Creatinine | 38(20) | 10(15) | 5 |
| INR | 75(39) | 20(31) | 8 |
| Total bilirubin | 74(38) | 20(31) | 7 |
| Dobutamine | 128(66) | 48(74) | -8 |
| Norepinephrine | 56(29) | 16(25) | 4 |
| **Baseline characteristics** | | | |
| Weight (Kg) | 39 (20) | 10 (15) | 5 |
| Days of ARDS to OLB | 2 (1) | 0(0) | 1 |
| Days of ADRS to hospital admission | 18(9) | 3(5) | 4 |
| Length of hospital stay | 26(13) | 6(9) | 4 |
| Days on invasive mechanical ventilation | 34(18) | 8(12) | 6 |

All variables are reported as n (%) of missing data
