# Supplementary figures and images for: Predicting the Impact of Diffuse Alveolar Damage through Open Lung Biopsy in Acute Respiratory Distress Syndrome—The PREDATOR Study
Source: J Clin Med. 2019 Jun 11;8(6):829. doi: 10.3390/jcm8060829 (PMC6616523; doi:10.3390/jcm8060829)

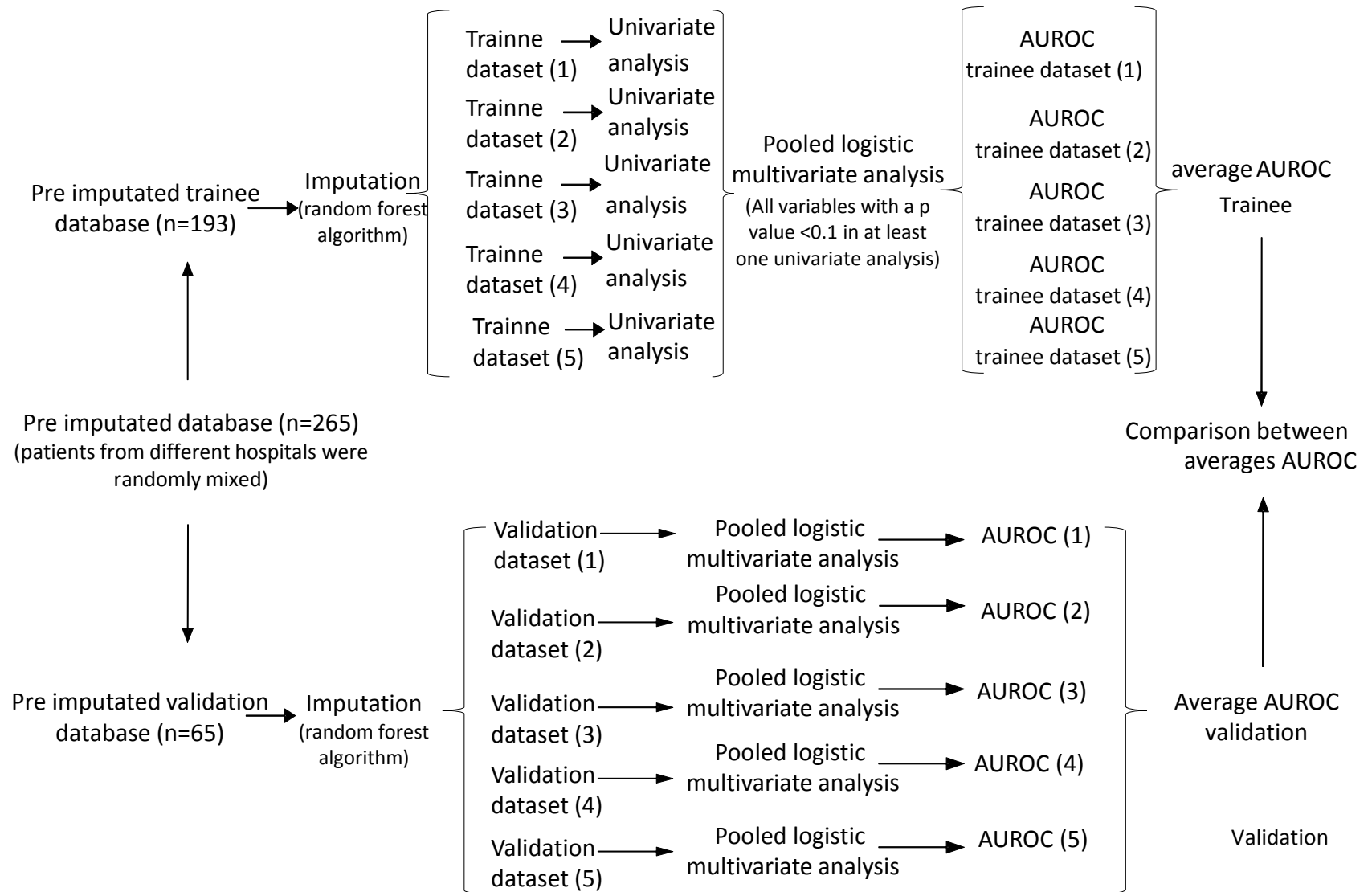

Figure S1

Supplement: Supplementary file 1 [file jcm-08-00829-s001.zip › Article Predator_Figure S1_V7 imputation algorithm.pdf]
